# A Case Report Examining Early Extubation Following Congenital Heart Surgery in a Low Resource Setting

**DOI:** 10.3389/fped.2018.00311

**Published:** 2019-03-19

**Authors:** Catherine Howes

**Affiliations:** Cardiac Intensive Care Unit, Great Ormond Street Hospital for Children, London, United Kingdom

**Keywords:** early, extubation, fast-track, surgical, mission, charity, paediatric, cardiac

## Abstract

This case report aims to critically analyse the evidence surrounding early extubation in the post-operative phase following complex congenital cardiac surgery. Child A was an 8 year old female who had undergone complex congenital cardiac surgery during an international surgical charity mission. On admission to the paediatric intensive care unit Child A appeared to be in good condition and no major complications had occurred intra-operatively. This was considered alongside the situational pressures of resource limitations and the mission's aim to offer surgery to as many children as possible during the available time frame. The decision was made by the team that Child A was a suitable candidate for ‘early extubation.’ Some members of the team were uncomfortable with this approach and felt it could lead to poorer outcomes for patients. Current evidence surrounding early extubation both within international surgical mission trips to low-income and middle-income countries and established cardiac centres within high-income countries is examined and discussed alongside the context of resource limitation. Although the process and implications of early extubation following cardiac surgery needs further research, on the basis of the evidence currently available clinicians could potentially encourage the use of early extubation within clinical practice (for appropriately selected patients) through the utilisation of a multidisciplinary approach, both within the UK and during international surgical charity missions to low-income and middle-income countries.

## Introduction

This case report aims to critically analyse the evidence surrounding early extubation in the postoperative phase following complex congenital cardiac surgery. The child [identified only as ‘Child A’ in order to conform to NMC guidelines (1)] was an 8 year old female who had undergone complete tetralogy of Fallot repair during an international surgical charity mission. Child A had no previous intervention and her tetralogy was deemed severe, with surgery more complex than classically seen in the UK, requiring a prolonged period on cardiopulmonary bypass and placement of an extensive transannular patch. Tetralogy of Fallot is the most common cyanotic congenital cardiac defect (2). It is characterised by the presence of four cardiac abnormalities - a malaligned VSD, overriding aorta, pulmonary stenosis, and right ventricular hypertrophy (3). Children usually require surgical intervention within the first six months of life in order to minimise long term morbidity and promote growth and development (4).

Primary complete repair in infancy is the current preferred surgical approach for the treatment of tetralogy of Fallot (5). However, a late presentation of tetralogy of Fallot is not uncommon in low-income and middle-income countries (and therefore during ‘surgical mission trips’), and a lack of surgical facilities may delay treatment further. As a result these ‘older’ children develop a number of significant risk factors including chronic hypoxaemia, polycythaemia, stunted growth, and significant left ventricular dysfunction. All of these risk factors were present in Child A - particularly stunted growth as a result of the underlying cardiac condition and malnutrition [often present in this group of patients as a result of living conditions and socioeconomic status (6)].

However, despite the presence of these risk factors there are a number of documented case series' where ‘late’ tetralogy of Fallot repair has been undertaken during later childhood in children from low-income and middle-income countries (with surgery completed in both the country of origin during surgical missions or pre-operative transfer of the child to an established cardiac centre in a high-income country). These show no difference in post-operative morbidity and mortality compared with children who undergo a standard infant repair, alongside fewer ventilation days and earlier discharge seen in the late repair group (6, 7).

Child A appeared to be in good condition on admission to the paediatric intensive care unit (PICU) post-operatively (run collaboratively between the local team and the international visiting team), maintaining appropriate vital signs, minimal bleeding and requiring minimal ventilation to achieve normal arterial blood gases. No major complications had occurred intra-operatively. This was considered alongside the situational pressures of resource limitations and the missions aim to offer surgery to as many children as possible during the available time frame. The decision was made by the team that Child A was a suitable candidate for ‘early extubation’ and extubation occurred within two hours of her admission to the PICU. Some members of the team were uncomfortable with this approach and felt it could lead to poorer outcomes for patients.

## Background

In recent years there have been significant advances in surgical, interventional cardiac catheterisation and hybrid procedures as treatments for congenital heart disease. Despite this, there are wide disparities in the treatment available for children around the world. It has been suggested that approximately 90% of children with congenital heart disease in low-income and middle-income countries do not have adequate access to essential treatment (8). This inequality in service provision is often due to the absence of a range of resources including lack of appropriately skilled paediatric cardiologists and cardiac surgeons, absence of suitable paediatric intensive care facilities, economic restraints and inadequate local infrastructure within low-income and middle-income countries.

In an attempt to bridge this gap, a number of international charities organise regular ‘surgical mission trips’ to low-income and middle-income countries across the world. These aim to provide services and education for areas of the world where access to treatment is limited by local health resources and/or cost, with the ultimate goal being to improve access and sustainability of local congenital heart disease programmes in low-income and middle-income countries (9). Whilst a number of different models of education and care exist within this context (10), all acknowledge that development of these programmes will take an extended period of time and frequent visits will be required. The ultimate aim is that the ‘mission trips’ will become redundant as local teams develop the skills and infrastructure required to run autonomous, self-sufficient programmes for children with congenital heart disease (11).

## Discussion

Within high-income countries (where paediatric cardiac surgery is generally accessible to all children) most surgical techniques developed to treat congenital heart disease involve the use of cardiopulmonary bypass. In order to facilitate this, intubation and mechanical ventilation are required and this is often continued during the post-operative period (12). The majority of children will be admitted to PICU intubated and ventilated for a period of time (often at least overnight) post cardiac surgery (13). The benefits of extubating children as soon as clinically possible is not new, having first been discussed in the literature in the 1970's (14). Early extubation following cardiac surgery in adults was introduced into standard clinical practice as early as the 1990's (15, 16). Since then, ‘fast track’ strategies have been developed in both populations, made up of four components: early extubation, early ambulation, cardiac rehabilitation, and early discharge (12).

Although ‘early extubation’ [extubation within the operating theatre or less than eight hours after a child's admission to PICU (17, 18)] has been described in the literature (19, 20), mechanical ventilation following cardiac surgery in children remains common practice (21). However, on reviewing the limited quantity of readily available literature, there is support for early extubation of appropriate patients. Large studies within adult cardiac surgery have clearly demonstrated that early extubation and ‘fast-tracking’ can be achieved safely and may be beneficial to the patient (22).

Harris et al. (23) performed a retrospective analysis on all neonatal and paediatric patients post cardiac surgery (within an established high-income centre) and concluded that early extubation led to a reduced length of stay in both PICU and hospital and correlated with low morbidity within the patient group. Other studies (24–26) also suggest that early extubation is safe and desirable and the benefits of early extubation are discussed including reduced risk of complications related to prolonged ventilation. It is clear that successful extubation of appropriately selected patients reduces the potential morbidity and mortality risks which are directly related to intubation and/or mechanical ventilation, including accumulation of respiratory secretions, atelectasis, nosocomial infections (including ventilator acquired pneumonia), airway trauma and unplanned extubation (27). Extubation may also remove or significantly reduce the need for continuous sedation and the inevitable undesirable side effects which often include respiratory and hemodynamic depression, sedation tolerance (requiring escalating doses), delirium and withdrawal once sedation is discontinued (27).

Further studies and case reports have suggested that early extubation in children following cardiac surgery can be achieved without an increase in post-operative complications, or adverse effects on cardiac function (17, 28). Within PICU in established cardiac centres in high-income countries, early extubation is actively encouraged for some post-operative cardiac conditions where extubation is physiologically favourable (i.e. Fontan procedures) and has become ‘standard’ clinical practice. Studies show that within these subgroups the majority of children are extubated within a few hours of admission to PICU compared to the minority of children following tetralogy of Fallot repair (29). It is interesting to note that no literature was found which suggested early extubation (for appropriately selected patients) led to poor outcomes or increased morbidity and/or mortality.

It is important to appreciate that a significant proportion of the literature surrounding early extubation in this field of practice comes from high-income countries where children are treated in well-established cardiac centres which face different pressures when compared to a surgical mission. When the literature referring to low-income and middle-income countries is explored, it is evident that early extubation is seen as a safe and often preferable practice within these environments (30). Akhtar et al. (31) suggest that early extubation in their group of patients resulted in improved patient outcomes and improved utilisation of available resources as a result of increased patient turnover from the PICU. This allowed the team to treat more patients in the limited timeframe available. Early extubation has shown to be cost effective in low-income and middle-income countries with limited health care resources (18), which is a significant factor to take into consideration when planning surgical missions within countries with a seemingly endless patient population (27). It is clear that some high risk groups are not appropriate candidates for early extubation. It is also important to recognise the difficulties in directly comparing patient groups seen in high-income countries (which see the full range of children requiring cardiac surgery) and mid/low-income countries and mission trips which often focus on lower risk patients (who may present with additional risk factors not commonly seen in high-income countries).

In the case of Child A it may be that early extubation was in her best interests. A late complete tetralogy of Fallot repair in an older child may result in the occurrence of restrictive right ventricular physiology during the initial post-operative period more frequently than if the repair is completed electively during infancy due to ventricular hypertrophy and fibrosis (32). If clinically significant, left ventricular filling may also be indirectly affected (33). In this scenario it is fundamentally important to optimise right ventricular preload - this can be supported by decreasing intrathoracic pressures through extubating the child and discontinuing mechanical positive pressure ventilation at the earliest opportunity. The change from positive pressure ventilation to spontaneous ventilation enhances cardiovascular function by reducing right ventricular afterload and improves preload (6, 27). Studies have shown that for post-operative patients following a complete tetralogy of Fallot repair, cardiac output and cerebral oxygenation increased significantly once extubation took place and spontaneous ventilation was re-established (34). Early extubation in children following a complete tetralogy of Fallot repair in low-income and middle-income countries with limited health care resources has shown to be safe and effective, as in the case of Child A where early extubation was not associated with complications (35).

Contrary to the assumption that late repair of tetralogy of Fallot in an older child would potentially result in an increase in morbidity and or mortality when compared to elective repair as an infant, studies have shown this is not the case and that no significant difference is evident (6). Although surgical decision making and intraoperative events play a significant role in the feasibility of extubating patients soon after surgery, another area of clinical practice which has a significant impact is anaesthesia. The anaesthetic team play a fundamental role in the planning and delivery of early extubation in the form of premedication, induction technique, intraoperative anaesthetic agents, neuromuscular blockade reversal and post-operative analgesia (18, 36, 37).

The development of new and improved anaesthetic agents including inhalational anaesthetics, short-acting opioids, hypnotics and sedatives with favourable pharmacodynamics (particularly with respect to depression of cardiac function) make the concept of early extubation following cardiac surgery possible if the process of anaesthesia is both well planned and well managed in appropriately selected patients (17). As these drugs and anaesthetic techniques become more readily available in low-income and middle-income countries (or can be provided by the visiting team), early extubation of paediatric cardiac patients can be achieved safely during surgical missions (31). It has been suggested that alongside improved surgical and bypass techniques, the most important factor influencing the success of early extubation is adequate provision of effective analgesia (12). This includes the use of non-opioid drugs such as paracetamol and the use of local anaesthetic (38).

It is clear that teams travelling to low-income and middle-income countries in order to take part in surgical missions are likely to face resource limitations, particularly if working within a centre that does not ordinarily have the infrastructure for cardiac surgery or paediatric intensive care. It is well-documented within the literature that surgical missions often face the problem of limited resources not usually evident within clinical practice in established cardiac centres within high-income countries (39, 40). Cardiac surgery is known to be a technologically dependent area of clinical practice with a reliance on single use consumables (41). This was recognised as early as the 1980's with the development of the ‘KISS (Keep It Simple and Safe)’ approach which aimed to provide treatment to the maximum number of patients within the context of limitations of funding, equipment and manpower (42). Whilst some degree of technology is essential for even the most basic cardiac surgery, international teams will likely find resource limitations a constant challenge. Welling et al. (43) identify ‘failing to match technology to local needs and abilities’ as one of their ‘Seven Sins of Humanitarian Medicine.’

It is also important to recognise that within the PICU where child A was admitted, parents were not allowed to visit. Whilst this is different to ‘standard’ clinical practice within the UK, the local policies and customs were respected as far as possible during the surgical mission. Regular updates were given to the parents by the local team and this appeared to satisfy them. It is recognised that parents from deprived backgrounds whose child is admitted to PICU experience significantly higher stress levels and this must be considered (44). Within the UK, invasive procedures including extubation may further increase parents stress levels (45, 46) (particularly if parents are aware there is a deviation from ‘usual’ clinical practice and is referred to as ‘early’ by staff). If integrated into clinical practice, parents must be informed and involved in all aspects of their child's care. However, the opposite has also been suggested in that early extubation and fast track strategies reduce parent anxiety if a child is extubated rapidly as this allows verbal communication with their child and earlier mobilisation (12, 31).

It is clear that patient selection at the time of surgical listing must carefully consider a wide variety of factors including patient safety, anticipated time to discharge back to local services, educational objectives and resource availability within the time constraints of the surgical mission. Teamwork and communication (including both the local and visiting teams) is crucial to all aspects of a surgical mission, but vital if early extubation of children following cardiac surgery is to be implemented. It is also important for the team to understand that even if a child is identified as a candidate for early extubation following their pre-operative assessment or at the time of surgical listing, early extubation cannot be guaranteed and is dependent on many factors including intra-operative and post-operative events and clinical progress (18). Despite substantial evidence showing that early extubation in children following surgery for congenital heart disease can be achieved safely, significant individual and institutional concerns about integrating the approach within clinical practice remain (31, 47).

Inevitably, challenges will arise for both the local and visiting teams during their collaborative practice. These must be dealt with in a sensitive manner so that no member of the team feels their practice is in anyway substandard or could lead to poorer outcomes for patients. The international team must be aware that significant adjustments to what is perceived as ‘standard’ clinical practice may have to be made in order to function as an effective team within a resource limited environment (11).

## Concluding Remarks

Upon reflection on this case, current knowledge suggests that early extubation following complex congenital heart surgery may not result in poorer outcomes for children if patients are selected and managed appropriately. Early extubation may in fact be beneficial for patients and lead to a faster recovery for children regardless of their underlying cardiac defect or the environment in which treatment is received. It has also been suggested that early extubation is a safe and effective method of reducing patient morbidity, improving patient turnover by accelerating a child's discharge from the PICU and has been shown to be a cost effective practice (12, 20). Studies have strongly supported the development of early extubation pathways for children undergoing congenital cardiac surgery in both low, middle, and high-income countries (17). As no large studies endorsing any potential benefits of early extubation have been undertaken, concerns about the practice remain an issue within daily clinical practice (47). It is clear that some high risk patient groups are not appropriate candidates for early extubation, particularly following highly complex surgery not commonly undertaken during mission trips. Although the process and implications of early extubation following cardiac surgery need further research, on the basis of the evidence currently available clinicians within the UK could potentially encourage the use of early extubation within clinical practice for appropriately selected patients through the utilisation of a multidisciplinary approach, both within the UK and during surgical missions.

## Consent

Oral parental consent for the discussion of this case was obtained.

## Author Contributions

The author confirms being the sole contributor of this work and has approved it for publication.

### Conflict of Interest Statement

The author declares that the research was conducted in the absence of any commercial or financial relationships that could be construed as a potential conflict of interest.
